# Ferulic acid and N-Feruloylserotonin ameliorate LPS-induced intestinal inflammation via modulation of gut microbiota, metabolome, and transcriptome

**DOI:** 10.3389/fmicb.2025.1597774

**Published:** 2025-07-22

**Authors:** Xiangdong Hu, Xuebing Han, Gang Liu, Guiping Guan, Chenmei Xia

**Affiliations:** ^1^Department of Gastroenterology, The First People’s Hospital of Wenling, Wenling, China; ^2^Hunan Provincial Engineering Research Center of Applied Microbial Resources Development for Livestock and Poultry, College of Bioscience and Biotechnology, Hunan Agricultural University, Changsha, China; ^3^State Key Laboratory of Subtropical Silviculture, Zhejiang A&F University, Hangzhou, China; ^4^Bamboo Diseases and Pests Control and Resources Development Key Laboratory of Sichuan Province, College of Life Science, Leshan Normal University, Leshan, China

**Keywords:** ferulic acid, N-Feruloylserotonin, LPS, intestinal microorganisms, metabolites, transcriptome

## Abstract

Intestinal homeostasis depends critically on the dynamic interplay between gut microbiota, epithelial barriers, and host immunity, dysregulation of this triad can initiate inflammatory cascades. Ferulic acid and its derivative N-Feruloylserotonin demonstrate significant anti-inflammatory activity, though their intestinal protective effects and mechanisms require further elucidation. Therefore, this study examined how these compounds mitigate lipopolysaccharide (LPS)-induced acute inflammation through integrated modulation of the gut microbiome, serum metabolome, and transcriptional networks. Our findings reveal that both compounds, attenuated LPS-induced intestinal pathology in murine models, suppressed pro-inflammatory cytokine expression, elevated beneficial metabolites including 1-naphthalenesulfonic acid, enriched probiotic taxa (Ruminococcaceae, Muribaculaceae, Lachnospiraceae, Bifidobacteriaceae, Prevotellaceae, Roseburia, Blautia, and Butyricicoccus), and suppressed pathobionts (Proteobacteria, Gammaproteobacteria, Enterobacterales, and Bacillus). Transcriptomic profiling further implicated modulation of antigen processing and presentation, NF-κB signal pathway, MAPK signal pathway, and PI3K-Akt signal pathway. Key regulatory targets identified include: Pik3cd, H2-DMb1, H2-Oa, Kdr, Fgfr3, Il1r2, Rac, Irak4, Traf6, Ticam1, Rip1, and Rip3. This work establishes a mechanistic foundation for deploying ferulic acid and N-Feruloylserotonin in intestinal health preservation and inflammatory disease prevention, while providing novel insights into microbiota-homeostasis crosstalk.

## Graphical abstract

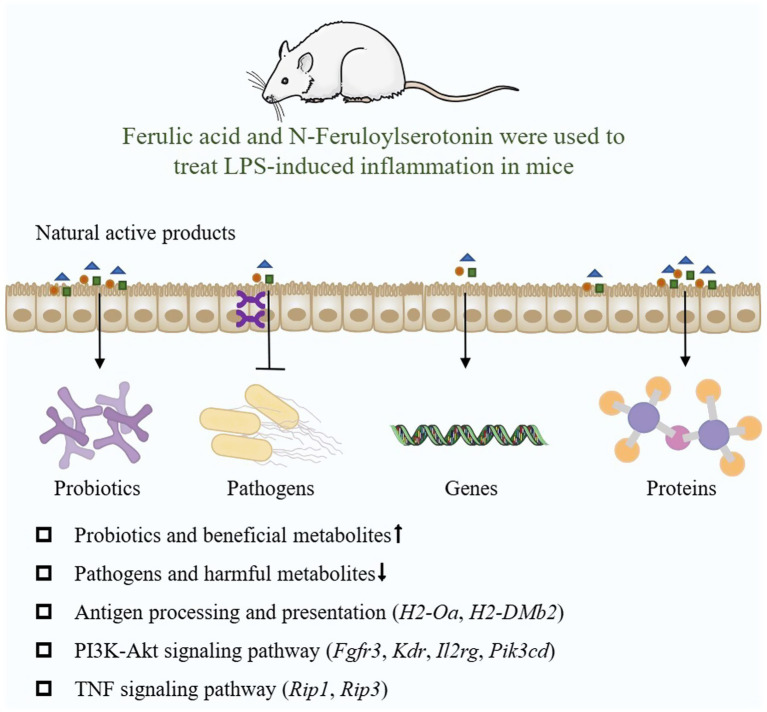

## 1 Introduction

The intestine constitutes the body’s largest immune organ, functioning not only in nutrient absorption but also as a critical physicochemical barrier against diverse pathogenic insults (Funk et al., 2020). Its innate immune system serves as the primary defense against enteric pathogens (Roh et al., 2021), with 70% of systemic immune cells residing in the gut mucosa to combat environmental toxins. Homeostasis is maintained through dynamic interactions between the gut microbiota and intestinal barrier, a balance essential for gastrointestinal health (Veiga-Fernandes and Pachnis, 2017). This crosstalk establishes an immunoregulatory microenvironment where immune cells coordinate with commensal flora to sustain mucosal immunity (Wang et al., 2023). Disruption of this equilibrium triggers systemic inflammation and predisposes to multiple pathologies, including neurodegenerative disorders (Loh et al., 2024), colorectal cancer (Li et al., 2024), and inflammatory bowel disease (Danne et al., 2024). Consequently, intestinal integrity is fundamental to metabolic regulation, immune competence, and pathogen resistance.

Natural products, bioactive compounds derived from living organisms, demonstrate anticancer, anti-inflammatory, antioxidant, and neuroprotective properties, offering therapeutic potential for disease prevention and management (Chopra and Dhingra, 2021). Their structural complexity and prolonged intestinal residence facilitate bidirectional interactions: they modulate microbial diversity while gut microbiota metabolize them into physiologically active compounds inaccessible to host biosynthesis (Zhao et al., 2022). Ferulic acid is a phenolic substance that is usually present in cereal seeds, grapes, parsley, whole grains, rhubarb, and spinach. It exhibits many biological activities, such as clearing excess ROS, free radicals, and enzymes that produce free radicals, thereby resisting oxidative damage and reducing inflammatory reactions (Li et al., 2021). In addition, ferulic acid also exerts anti-inflammatory effects by regulating PPAR γ, NF-κB, and MAPK signaling pathways, which has a protective effect on various diseases (Li et al., 2021). Previous studies have documented the protective effects of ferulic acid on the intestinal tract (Hwang et al., 2022; Tian et al., 2022); however, a comprehensive assessment of its mechanism of action, impact on gut microbiota, and associated metabolomic alterations remains lacking. N-Feruloylserotonin is an amide formed by 5-hydroxytryptamine and ferulic acid, which is a derivative of ferulic acid and widely present in many plants (Carola et al., 2021). N-Feruloylserotonin has many anti-inflammatory functions similar to ferulic acid, which can significantly reduce the production of reactive oxygen species, nitric oxide and prostaglandin E2 induced by LPS, and inhibit NF-κB signal pathway (Park et al., 2023). However, the intestinal protective effects of N-Feruloylserotonin and their underlying mechanisms remain unknown.

This study employed LPS-induced mouse acute intestinal injury to investigate both compounds. We assessed the structural protection via jejunal histopathology, microbial shifts through 16S rRNA sequencing, metabolic reprogramming via untargeted metabolomics and mechanistic pathways by transcriptomic profiling. Our findings elucidate how ferulic acid and N-Feruloylserotonin ameliorate intestinal inflammation, establishing a theoretical framework for their therapeutic application in gut health maintenance and inflammatory disease prevention. Furthermore, we provide novel insights into microbiota-homeostasis dynamics.

## 2 Methods

### 2.1 Animals

All experimental protocols received prior approval from the Biomedical Research Ethics Committee of Hunan Agricultural University (Approval No. 2023-51). Thirty-two 8-week-old male ICR mice were acclimatized in specific pathogen-free (SPF) environmental chambers maintained at 25 ± 3°C with 50 ± 5% relative humidity under standardized photoperiodic conditions (12-h light/dark cycle). Following a 7-day acclimatization period, mice were randomly allocated to four experimental groups (*n* = 8/group): vehicle group (CTRL); LPS-only group (LPS); LPS and ferulic acid treatment group (FA); LPS and N-Feruloylserotonin treatment group (NFS). Dietary composition and nutritional profiles are detailed in Supplementary Tables S1, S2. All experimental groups received the standard diet throughout the study. The acclimatization endpoint was designated as Day 0. Commencing on Day 1, the FA group received dietary supplementation of 200 mg/kg ferulic acid. From Day 22 onward, the NFS group was administered 5 mg/kg N-Feruloylserotonin via dietary incorporation. On the 27th day, the mice in the LPS, FA and NFS groups were intraperitoneally injected with 10 mg/kg LPS (from *E. coli* O55: B5, dissolved in water to prepare a stock solution of 10 mg/mL), and mice in the CTRL group were injected with the same volume of normal saline. Cervical dislocation was performed to euthanize the mice 24 h later. During the procedure, the operator grasped the base of the mouse’s tail firmly with the right hand and elevated the animal. The mouse was then positioned on a cage lid or other textured surface. The operator applied firm downward pressure to the head and cervical region using the thumb and index finger of the left hand. Concurrently, the tail base was grasped with the right hand and pulled sharply upward and backward. This action resulted in cervical dislocation, severing the connection between the spinal cord and the brainstem, leading to instantaneous death of the animal.

### 2.2 HE staining

First, the jejunal intestinal slices were harvested immediately post-euthanasia, rinsed in ice-cold saline, and processed fixed in 4% formaldehyde for HE staining. Then, the fixed slices were subjected to gradient dehydration with ethanol. Next, the dehydrated slices were embedded with paraffin. After hematoxylin and eosin staining, the tissue damage of mice in the four groups was observed and analyzed by microscope. Specific methods can refer to previous study (Han et al., 2023).

### 2.3 qPCR

The colon tissue was a separate aliquot was snap-frozen in liquid nitrogen and cryopreserved at −80°C for RNA extraction. The expression of tight junction protein genes, inflammatory cytokines, and specific marker molecules was detected by qPCR. After extracting RNA from mouse colon tissue, the concentration was measured using a spectrophotometer. Supplementary Table S3 is a list of primers (Gene-specific primer pairs for qPCR analysis were designed using NCBI, and synthesized commercially by Sangon Biotech). The reaction system includes “2 × SYBR Green Master Mix 10 μL, 0.5 μL of forward and reverse primers, 2 μL of cDNA template, ddH₂O supplemented to 20 μL,” and the reaction conditions are “pre-denaturation at 95°C for 3 min, followed by 40 cycles of 95°C for 15 s, 60°C for 30 s “. The specific methods and calculation formulas were referred to previous studies (Ren et al., 2014; Ma et al., 2019).

### 2.4 Intestinal microbial sequencing

After collecting the colonic contents of mice, the microbial DNA was isolated, and its concentration and purity were tested. Then, the V3-V4 region of 16S rRNA was amplified by primers F (5′-ACTCCTACGGGAGGCAGCA-3′) and R (5′-GGACTACHVGGGTWTCTAAT-3′). High throughput sequencing was carried out on Illumina platform after the purification of PCR products. Using SILVA as the reference database, the Naive Bayes Classifier was used to classify the feature sequences. Then, the data is filtered, with the filtering condition is set to base greater than 50% and mass greater than 20. The clean tags are clustered into OTUs with similarity greater than 97%. In order to detect the composition of community and α diversity of microorganisms, RDP classifier (v.2.2) and mothur (version 1.33.3) were used, respectively. α diversity metrics is primarily evaluated using indices such as Shannon index, Simpson index, Chao1 index, ACE index, and PD-whole-tree index. LEfSe was used to identify the species with different abundances.

### 2.5 Serum metabolites analysis

Blood samples were collected from the orbital sinus of mice using sterile technique. Ophthalmic forceps were employed to apply gentle pressure to the globe, facilitating the natural efflux of blood. The exudate was collected directly into pre-chilled EDTA-coated collection tubes. The blood samples were centrifuged for 10 min at the condition of 3,500 rpm, 4°C to collect the serum of mice. Then, LC–MS was performed in positive ion and negative ion modes respectively, and the raw data were obtained. This study employed a NMR platform. SIMCA14.1 (Umetrics, Umea, Sweden) was used to perform data statistics, with the serum metabolites were determined by comparing peak with metabolites in Human Metabolome Database. Pathway analysis, encompassing both enrichment analysis and topological analysis, was performed by integrating differential metabolites via the MetaboAnalyst 3.0 platform.

### 2.6 Transcriptome analysis

The purity, concentration and integrity of total RNA extracted from mice colonic tissue were detected. And the library was constructed as follows: The mRNA was randomly interrupted using a fragmentation buffer after the enrichment of mRNA. Then, the first cDNA chain and second strand were synthesized using mRNA as template. The purified double stranded cDNA was subjected to end repair, A-tail addition, and sequencing adapter connection, followed by fragment size selection using AMPure XP beads. Finally, the cDNA library was obtained by PCR enrichment. After the construction of the library, the Qsep400 high-throughput analysis system was used to detect the insert fragments of the library. In order to ensure the library quality, q-PCR was used to accurately quantify the effective concentration (>2 nM). After that, the sequencing was performed through Illumina NovaSeq6000 sequencing platform. After filtering to get clean data, the mapped data was obtained by sequence alignment with a specified reference genome. Finally, library quality evaluation, structure level analysis, differential expression analysis, gene function annotation and function enrichment were carried out. Finally, the library quality evaluation, differential expression analysis, gene function annotation and function enrichment were performed through BMKCloud (www.biocloud.net). Differentially expressed genes (DEGs) were analyzed by DESeq2 software. DEGs with |fold change| > 2 and Q value (adjusted *p*-value) < 0.05 were considered to be significantly different expressed genes.

### 2.7 Data analysis

The software SPSS 21 (SPSS, Inc., Chicago, United States), R and GraphPad were used to compare the differences among groups through one-way ANOVA with a Tukey HSD. The data were expressed as mean value ± standard deviation (mean ± SD). When *p* < 0.05, the difference between the groups was statistically significant.

## 3 Results

### 3.1 The effects of ferulic acid and N-Feruloylserotonin on intestinal injury and barrier of mice

**Figure 1:**
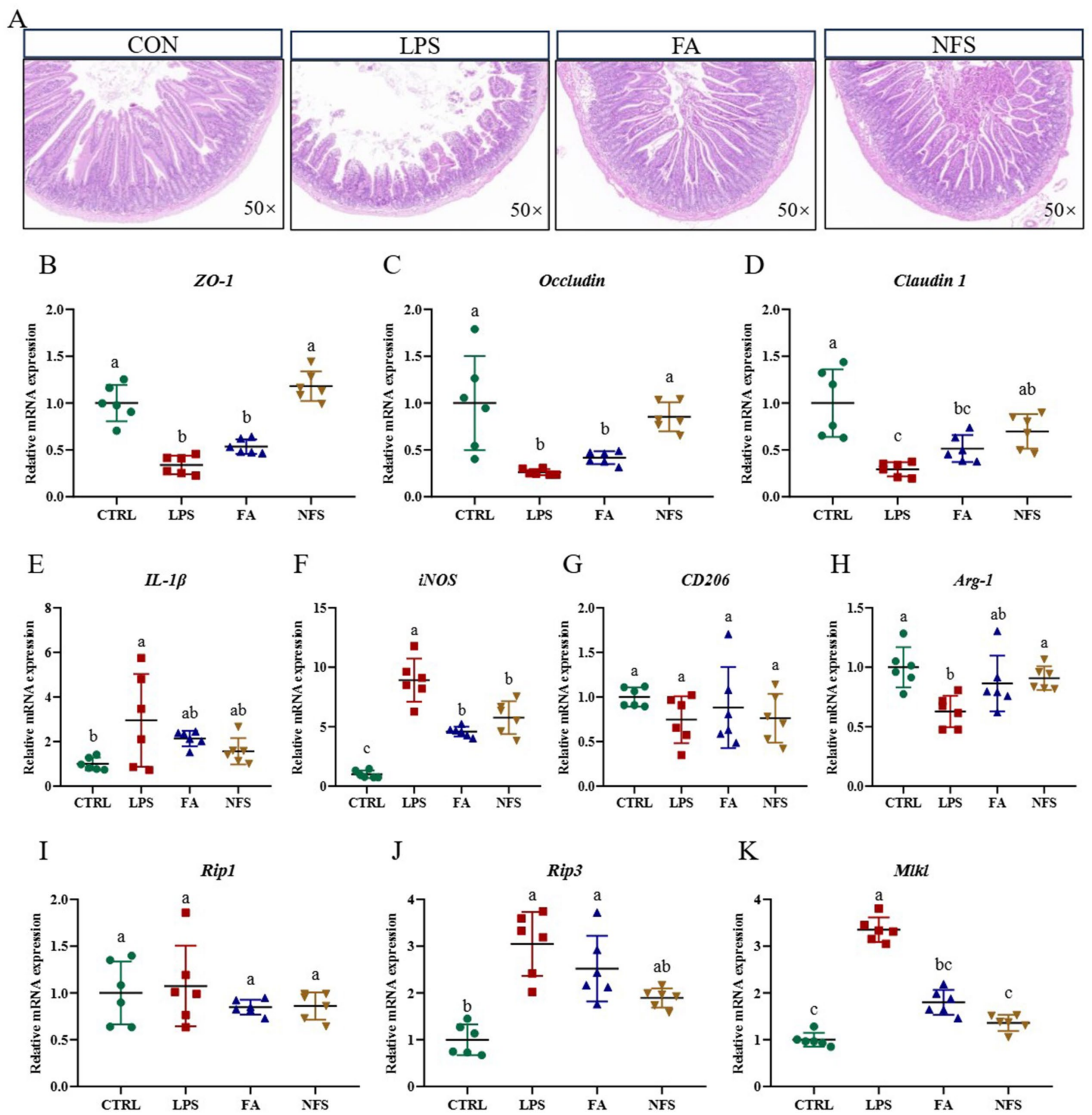
The effect of ferulic acid and N-Feruloylserotonin on the intestinal structure and barrier of mice. The images of jejunal tissue **(A)** and relative expression of *ZO-1* **(B)**, *Occludin* **(C)**, *Claudin 1* **(D)**, *IL-1β* **(E)**, *iNOS* **(F)**, *CD206* **(G)**, *Arg-1* **(H)**, *Rip1* **(I)**, *Rip3* **(J)**, and *Mlkl* **(K)**. Data are mean ± SD (*n* = 6). The absence of the same letter mark indicating significant differences (*p* < 0.05).

Histopathological analysis of jejunal tissue revealed intact mucosal architecture with tightly arranged villi in control (CTRL) mice. In contrast, LPS-challenged mice exhibited significant intestinal damage characterized by villous atrophy and epithelial denudation. Notably, both ferulic acid (FA) and N-Feruloylserotonin (NFS) treatments ameliorated these structural alterations (Figure 1A). Expression profiles of tight junction proteins differed significantly across groups (Figures 1B–D). LPS administration markedly suppressed *Claudin 1*, *Occludin* and *ZO-1* expression when compared with the CTRL group (*p* < 0.05). FA treatment partially restored expression levels, whereas NFS administration induced significant upregulation of all three genes (*p* < 0.05).

Pro-inflammatory cytokine *IL-1β* was significantly elevated in the LPS group (*p* < 0.05). Both compounds reduced *IL-1β* expression, though not statistically significantly (Figure 1E). Concurrently, LPS-induced *iNOS* expression, a downstream effector of proinflammatory signaling, was significantly attenuated by FA and NFS treatments (Figure 1F, *p* < 0.05). While *CD206* expression showed a non-significant reduction following LPS exposure, both compounds demonstrated restorative trends (Figure 1G). *Arg-1* expression was significantly suppressed in LPS-treated mice (*p* < 0.05), with both interventions normalizing expression to CTRL levels (Figure 1H).

Necroptosis-related gene expression in murine colonic tissue was assessed (Figures 1I–K). LPS challenge significantly upregulated *Rip3* and *Mlkl* expression (*p* < 0.05), with a concomitant non-significant increase in *Rip1*. Both ferulic acid and N-Feruloylserotonin interventions significantly downregulated *Mlkl* expression (*p* < 0.05), and reduced expression trends for *Rip1* and *Rip3*.

### 3.2 The effects of ferulic acid and N-Feruloylserotonin on intestinal microbiota in mice

**Figure 2:**
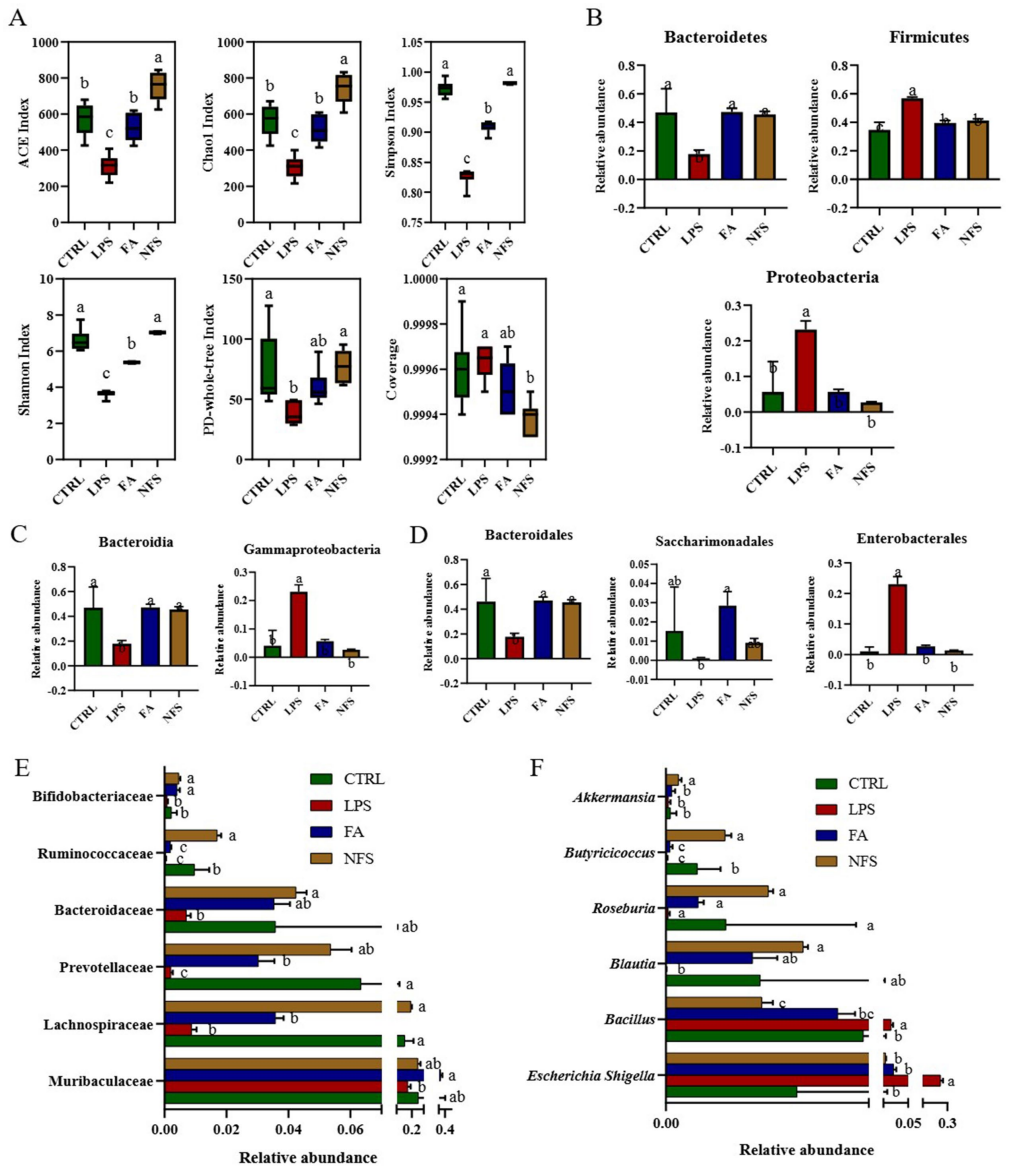
The effects of ferulic acid and N-Feruloylserotonin on intestinal microbiota in mice. The microbial α diversity of mice **(A)** and abundance of gut microbe phyla **(B)**, gut microbe classes **(C)**, gut microbe orders **(D)**, gut microbe families **(E)**, and gut microbe genera **(F)**. Data are mean ± SD (*n* = 6). The absence of the same letter mark indicating significant differences (*p* < 0.05).

Intestinal microbial diversity was quantified across experimental groups using α diversity metrics. As shown in Figure 2A, the LPS group exhibited significant reductions in Shannon index, Simpson index, Chao1 index, ACE index, and PD-whole-tree index (*p* < 0.05), whereas FA and NFS interventions significantly elevated these diversity parameters (*p* < 0.05).

Taxonomic profiling at the phylum levelrevealed marked alterations (Figure 2B). Bacteroidetes was inhibited significantly in the LPS group, and it was promoted significantly in the FA and NFS groups (*p* < 0.05). Proteobacteria and Firmicutes in the four groups showed an opposite trend to that of Bacteroidetes, which were significantly promoted by LPS and significantly inhibited by ferulic acid and N-Feruloylserotonin (*p* < 0.05). Many microorganisms have changed in different groups at the class level. Among them, the abundance of Bacteroidia in LPS group was significantly decreased, and the abundance of Gammaproteobacteria was significantly increased when compared with the CTRL group (Figure 2C, *p* < 0.05). Ferulic acid and N-Feruloylserotonin restored them to the similar level with CTRL group. As for the order level (Figure 2D), Bacteroidales in the LPS group was significantly inhibited, the abundance of Saccharimonadales decreased slightly, while Enterobacterales was promoted significantly (*p* < 0.05). After treatment with ferulic acid, Bacteroidales and Saccharimonadales showed significant increases in abundance, and Enterobacterales was inhibited significantly (*p* < 0.05). After N-Feruloylserotonin treatment, the abundance of Bacteroidales increased significantly, and Enterobacterales was also inhibited significantly (*p* < 0.05). At family level, the abundance of Lachnospiraceae, Prevotellaceae and Ruminococcaceae in LPS group decreased significantly, and they were significantly promoted by N-Feruloylserotonin, with Prevotellaceae was also promoted significantly by ferulic acid (Figure 2E, *p* < 0.05). Moreover, Muribaculaceae, Bacteroidaceae, and Bifidobacteriaceae were slightly decreased in LPS group, while ferulic acid significantly promoted the growth of Muribaculaceae and Bifidobacteriaceae, and N-Feruloylserotonin significantly promoted the growth of Bacteroidaceae and Bifidobacteriaceae (*p* < 0.05). *Butyricicoccus* at the genus level was inhibited significantly in the LPS group and promoted significantly in the NFS group (Figure 2F, *p* < 0.05). Moreover, the abundance of *Akkermansia, Roseburia*, and *Blautia* in the LPS group also tended to decrease, while N-Feruloylserotonin significantly increased that of *Blautia* and *Akkermansia* (*p* < 0.05). The abundance of *Bacillus* and *Escherichia Shigella* in LPS group increased significantly, while ferulic acid and N-Feruloylserotonin significantly reduced their abundance (*p* < 0.05).

**Figure 3:**
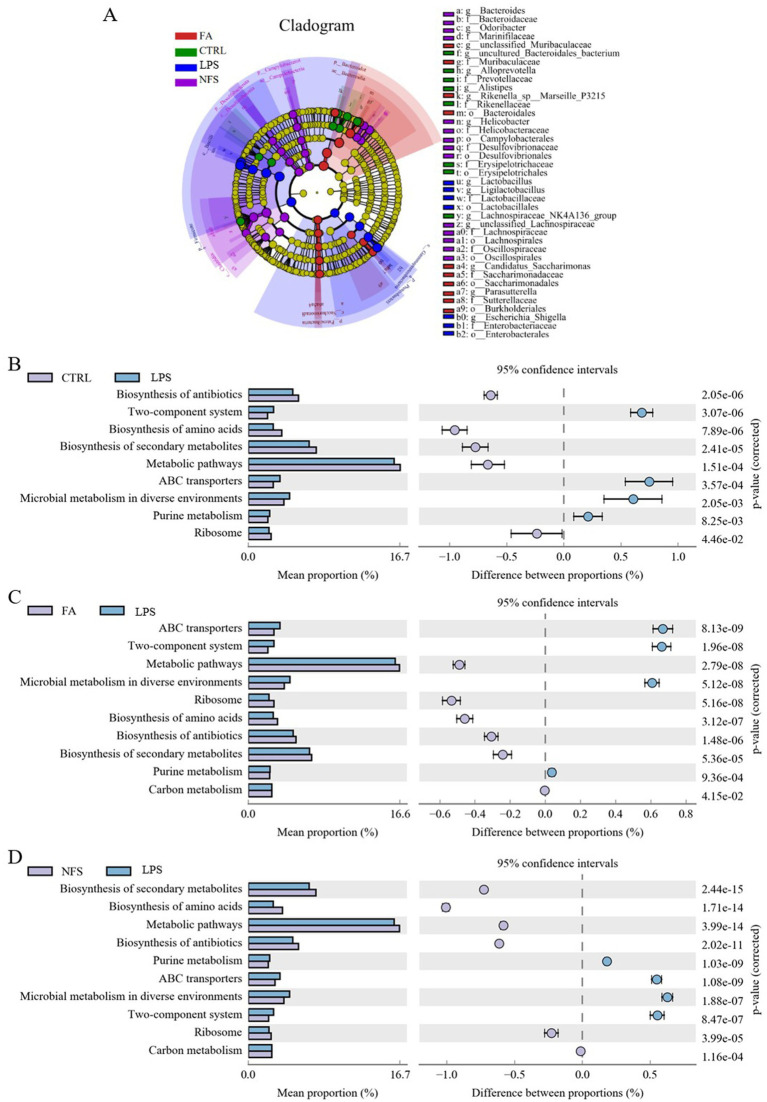
Changes in the composition and function of gut microorganisms. LEfSe **(A)** and significant differences in KEGG enrichment pathways in CTRL *vs* LPS groups **(B)**, FA *vs* LPS groups **(C)**, and NFS *vs* LPS groups **(D)**.

LEfSe analysis identified taxonomic biomarkers characteristic of each group (Figure 3A). The family Prevotellaceae, and Rikenellaceae, genus *Alloprevotella* and *Alistipes* were species with high abundance in the CTRL group. The phylum Firmicute and Proteobacteria, genus *Escherichia Shigella* were species with high abundance in the LPS group. The phylum Bacteroidetes, class Bacteroidia and Saccharimonadia, genus *Parasutterella* were species with high abundance in the FA group. The phylum Desulfobacterota, order Lachnospirales, genus *Bacteroides* were species with high abundance in the NFS group.

Functional gene enrichment analysis of colonic microbiomes revealed distinct pathway signatures (Figures 3B–D). The LPS *vs* CTRL comparison identified 9 significantly altered pathways, including microbial metabolism in diverse environments, biosynthesis of secondary metabolites, biosynthesis of antibiotics, two-component system, biosynthesis of amino acids, metabolic pathways, ABC transporters, purine metabolism, and ribosome. The significant differential enrichment pathways between FA and LPS groups were two-component system, microbial metabolism in diverse environments, biosynthesis of amino acids, metabolic pathways, biosynthesis of secondary metabolites, ribosome, biosynthesis of antibiotics, purine metabolism, ABC transporters, and carbon metabolism. The significant differential enrichment pathways between NFS and LPS groups were biosynthesis of secondary metabolites, biosynthesis of amino acids, metabolic pathways, biosynthesis of antibiotics, purine metabolism, ABC transporters, microbial metabolism in diverse environments, two-component system, ribosome, and carbon metabolism.

### 3.3 The effects of ferulic acid and N-Feruloylserotonin on serum metabolites in mice

**Figure 4:**
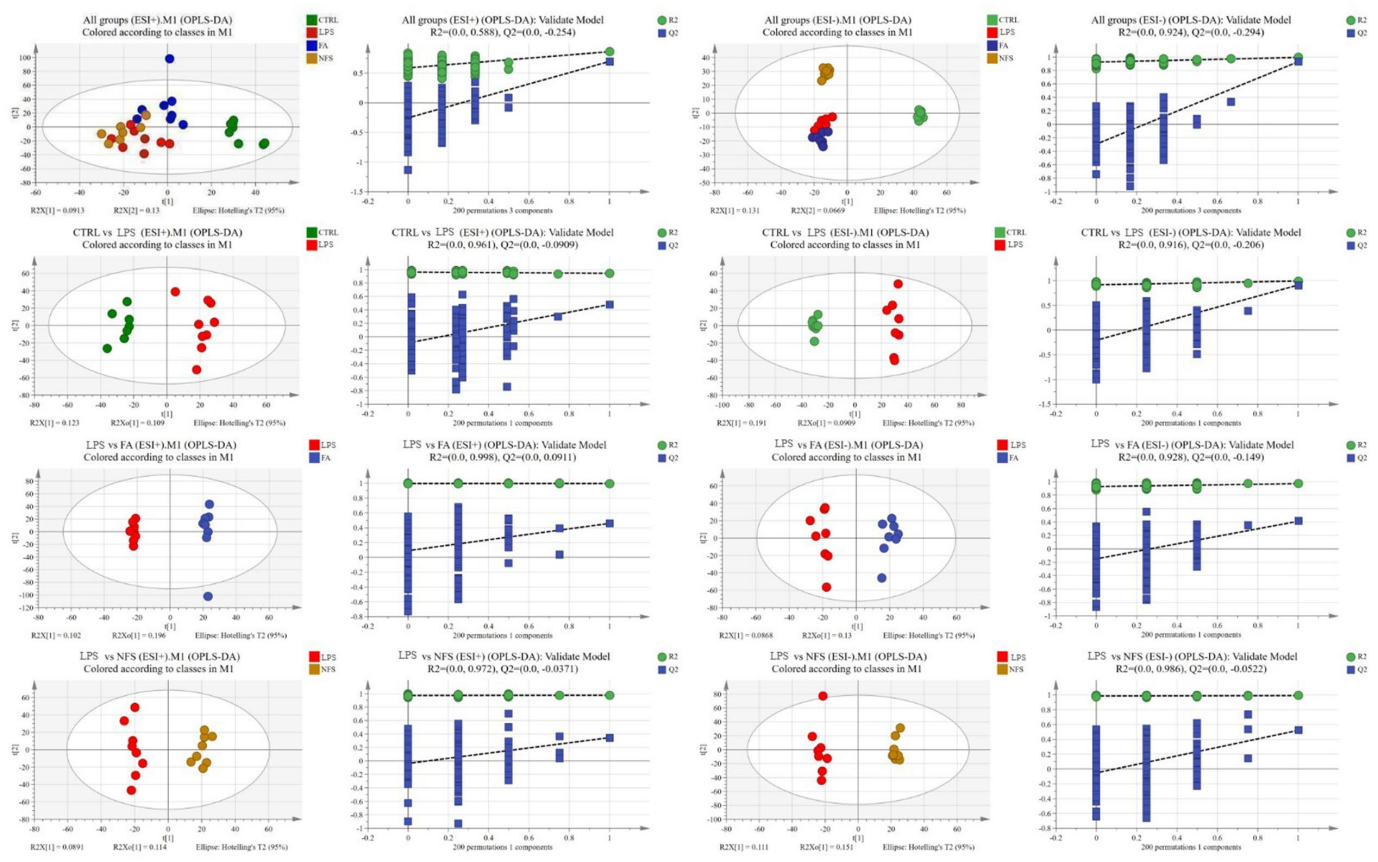
Multivariable statistical comparison plots of metabolites in groups.

Orthogonal partial least squares discriminant analysis (OPLS-DA) was employed to evaluate metabolic profiles across four experimental groups under both positive and negative ionization modes (Figure 4). The OPLS-DA score plots demonstrated complete separation of sample clusters between distinct groups, indicating statistically significant intergroup metabolic disparities, while intragroup sample aggregation reflected high metabolic homogeneity within each cohort.

**Figure 5:**
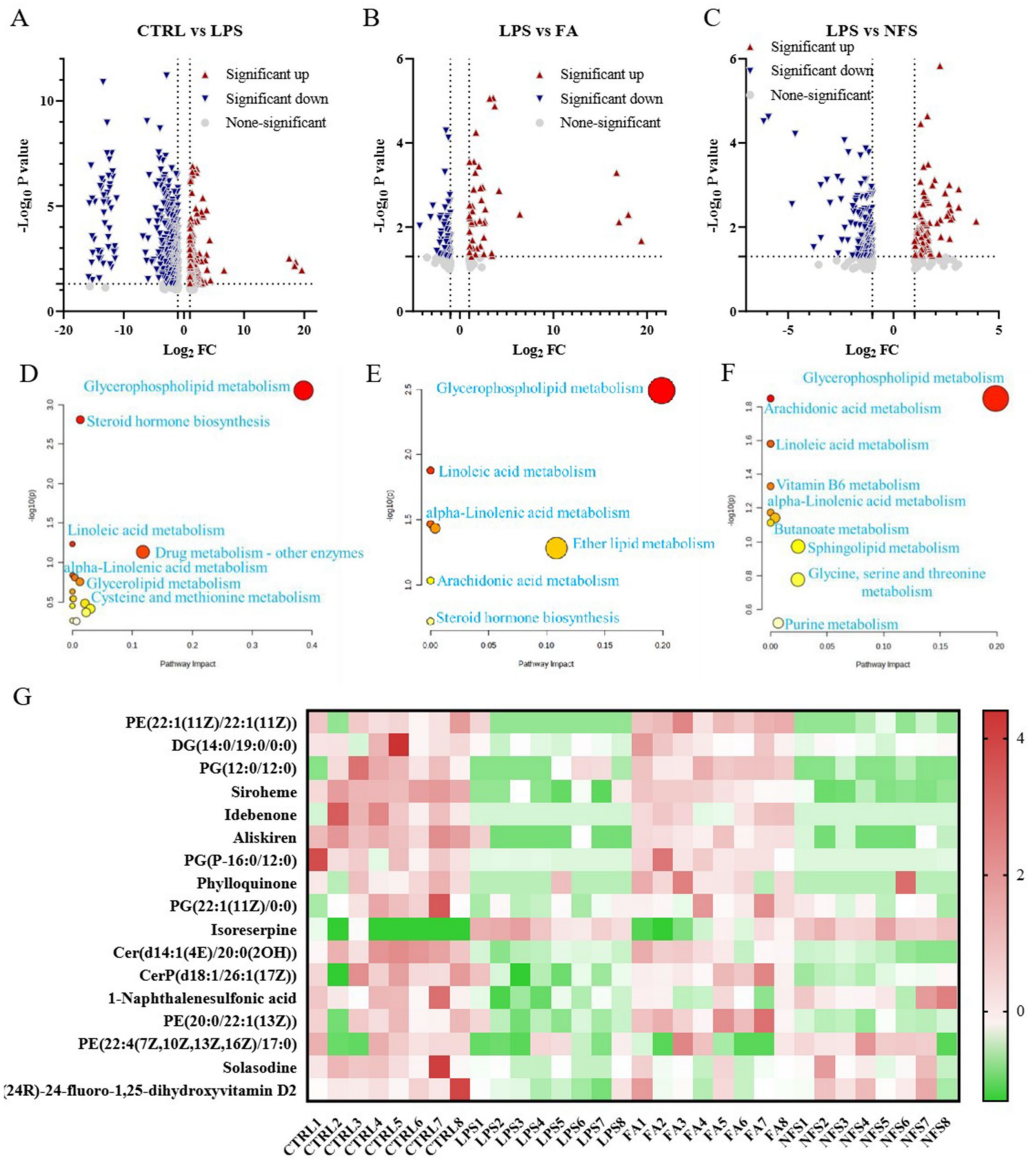
The effects of ferulic acid and N-Feruloylserotonin on serum metabolites in mice. Volcano maps of differential metabolites in CTRL *vs* LPS groups **(A)**, FA *vs* LPS groups **(B)**, and NFS *vs* LPS groups **(C)**; The enrichment pathway of differential metabolites in CTRL *vs* LPS groups **(D)**, LPS *vs* FA groups **(E)**, and LPS *vs* NFS groups **(F)**; Content of metabolites in different groups **(G)**.

Volcano plot visualization was utilized to identify differentially abundant metabolites in three comparative analyses: CTRL *vs* LPS, FA *vs* LPS, and NFS *vs* LPS (Figures 5A–C). Pathway enrichment analysis of significant metabolic alterations revealed conserved and group-specific perturbations. In CTRL *vs* LPS comparisons, alpha-linolenic acid metabolism, steroid hormone biosynthesis, linoleic acid metabolism, and glycerophospholipid metabolism constituted the primary enriched pathways (Figure 5D). The FA *vs* LPS comparison yielded identical pathway signatures (alpha-linolenic acid, glycerophospholipid, steroid hormone, and linoleic acid metabolism) (Figure 5E). Notably, the NFS *vs* LPS analysis expanded the pathway repertoire to include vitamin B6 metabolism and sphingolipid metabolism in addition to the conserved pathways (Figure 5F).

A hierarchical clustering heatmap was constructed to visualize metabolites exhibiting significant differential expression (Figure 5G). Compared with the CTRL group, PE(22:1(11Z)/22:1(11Z)), DG(14:0/19:0/0:0), PG(12:0/12:0), Siroheme, Idebenone, Aliskiren, PG(P-16:0/12:0), Cer(d14:1(4E)/20:0(2OH)), Phylloquinone, CerP(d18:1/26:1(17Z)), and 1-Naphthalenesulfonic acid were decreased significantly in the LPS group (*p* < 0.05). In the FA group, the content of PE(22:1(11Z)/22:1(11Z)), PG(12:0/12:0), Siroheme, Aliskiren, PG(P-16:0/12:0), and Cer(d14:1(4E)/20:0(2OH)) increased significantly (*p* < 0.05). PE(20:0/22:1(13Z)) was also slightly decreased in the LPS group, and upregulated by ferulic acid. Compared with the CTRL group, 1-Naphthalenesulfonic acid and Solasodine were significantly down-regulated in the LPS group (*p* < 0.05). In the NFS group, the content of 1-Naphthalenesulfonic acid increased significantly (*p* < 0.05), with the content of Solasodine also increased slightly. In addition, (24R)-24-fluoro-1,25-dihydroxyvitamin D2 and PE(22:4(7Z,10Z,13Z,16Z)/17:0) also showed a downward trend in the LPS group and were slightly up-regulated by N-Feruloylserotonin.

### 3.4 The effects of ferulic acid and N-Feruloylserotonin on colonic transcriptome in mice

**Figure 6:**
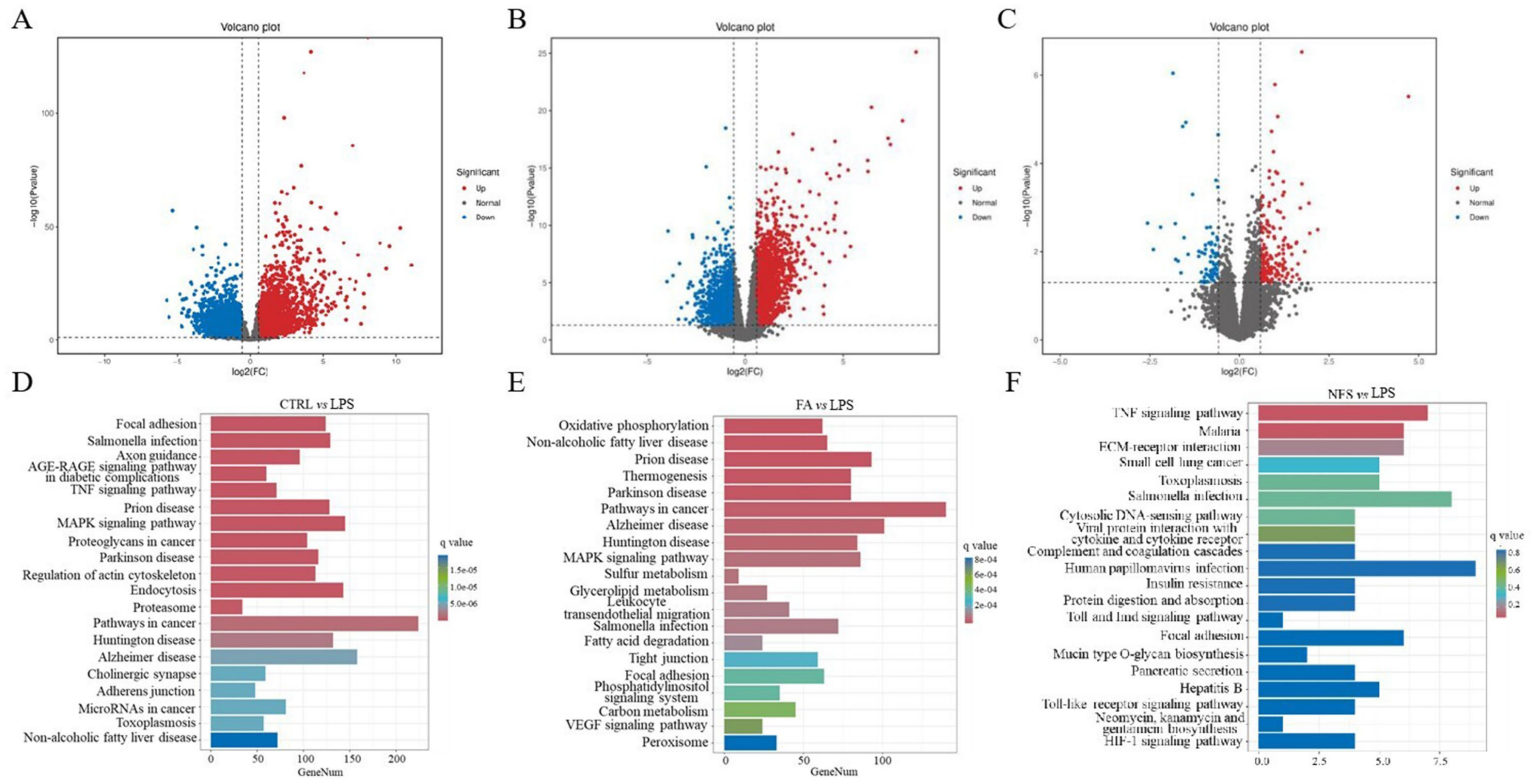
The effects of ferulic acid and N-Feruloylserotonin on colonic transcriptome in mice. Volcano maps of differentially expressed genes in CTRL *vs* LPS groups **(A)**, FA *vs* LPS groups **(B)**, and NFS *vs* LPS groups **(C)**; The enrichment pathway of differentially expressed genes in CTRL *vs* LPS groups **(D)**, LPS *vs* FA groups **(E)**, and LPS *vs* NFS groups **(F)**.

Volcano plots were generated to visualize DEGs across experimental comparisons (Figures 6A–C). A total of 6,324 DEGs were identified in the CTRL *vs* LPS comparison, 3,517 DEGs in the FA *vs* LPS comparison, and 248 DEGs in the NFS *vs* LPS comparison. Pathway enrichment analysis of these DEGs revealed distinct functional signatures (Figures 6D–F). In the CTRL *vs* LPS groups, the DEGs were mainly enriched in the TNF signaling pathway, MAPK signaling pathway, focal adhesion, and MicroRNAs in cancer. The main differential enrichment pathways of FA *vs* LPS groups included MAPK signaling pathway, glycerolipid metabolism, fatty acid degradation, tight junction, and VEGF signaling pathway. As for the differentially expressed genes in the NFS and LPS groups, they were mainly enriched in TNF signaling pathway, insulin resistance, Toll and Imd signaling pathway, focal adhesion, and HIF-1 signaling pathway.

**Figure 7:**
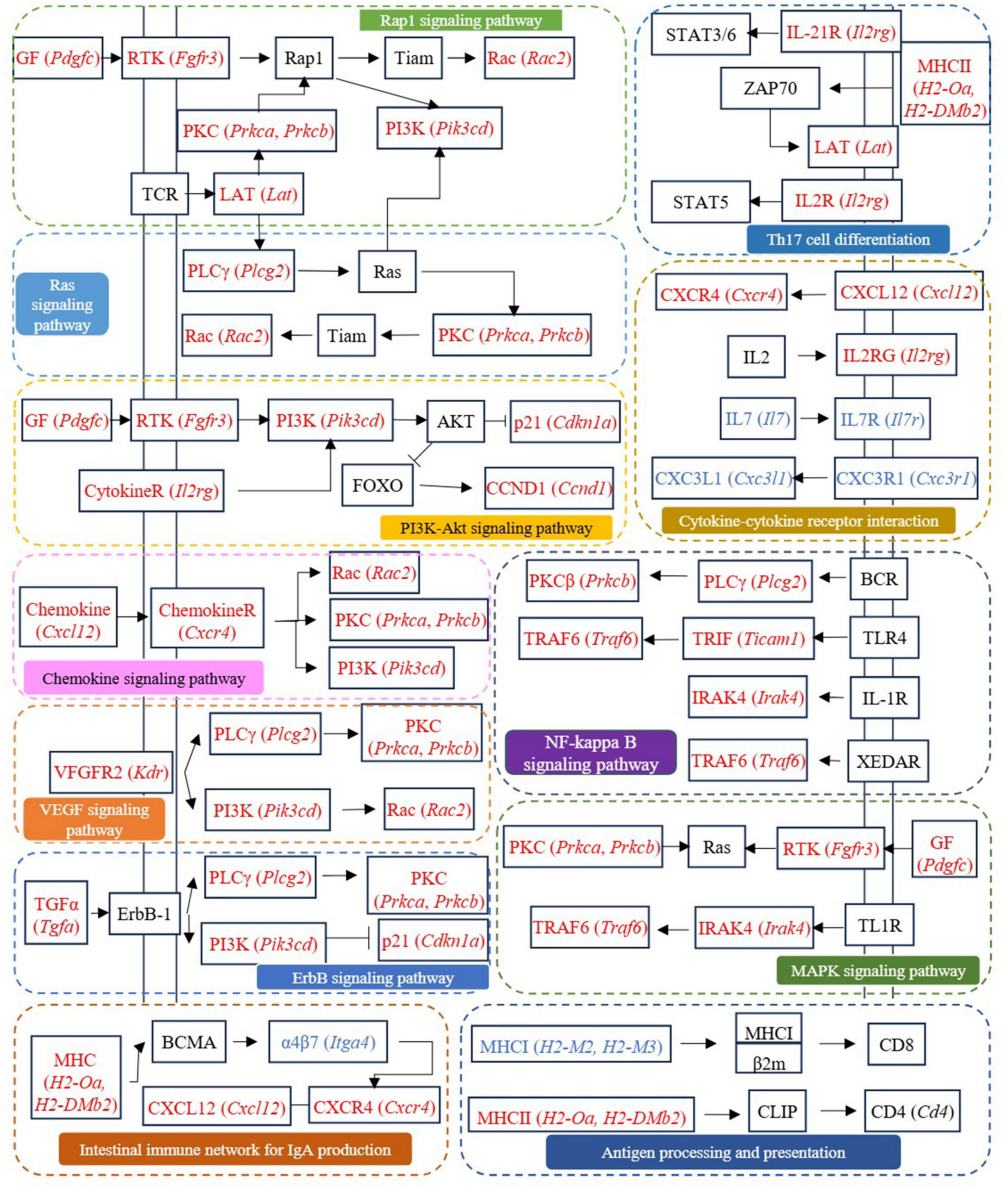
The signaling pathways involved in differentially expressed genes co-regulated by CTRL *vs* LPS and FA *vs* LPS groups.

Cross-comparison analysis identified 123 DEGs co-regulated in both CTRL *vs* LPS and FA *vs* LPS comparisons (|log₂FC| > 2), comprising 80 genes upregulated in LPS but downregulated with FA treatment, and 43 genes exhibiting reciprocal regulation patterns (Supplementary Table S5). Among these co-regulated genes, *Pik3cd*, *Prkca*, *Prkcb*, *Ccnd1*, *Cdkn1a*, *Plcg2*, *Rac2*, *H2-Oa*, *H2-DMb2*, *Tgfa*, *Cxcr4*, *Cxcl12*, *Il2rg*, *Pdgfc*, *Fgfr3*, *Ticam1*, *Irak4*, *Traf6*, and *Lat*, which involved in antigen processing and presentation, PI3K-Akt signaling pathway, Rap1 signaling pathway, Ras signaling pathway, Th17 cell differentiation, chemokine signaling pathway, VEGF signaling pathway, ErbB signaling pathway, MAPK signaling pathway, NF-kappa B signaling pathway, intestinal immune network for IgA production, and cytokine-cytokine receptor interaction, may be the potential targets for ferulic acid to regulate intestinal inflammation (Figure 7).

**Figure 8:**
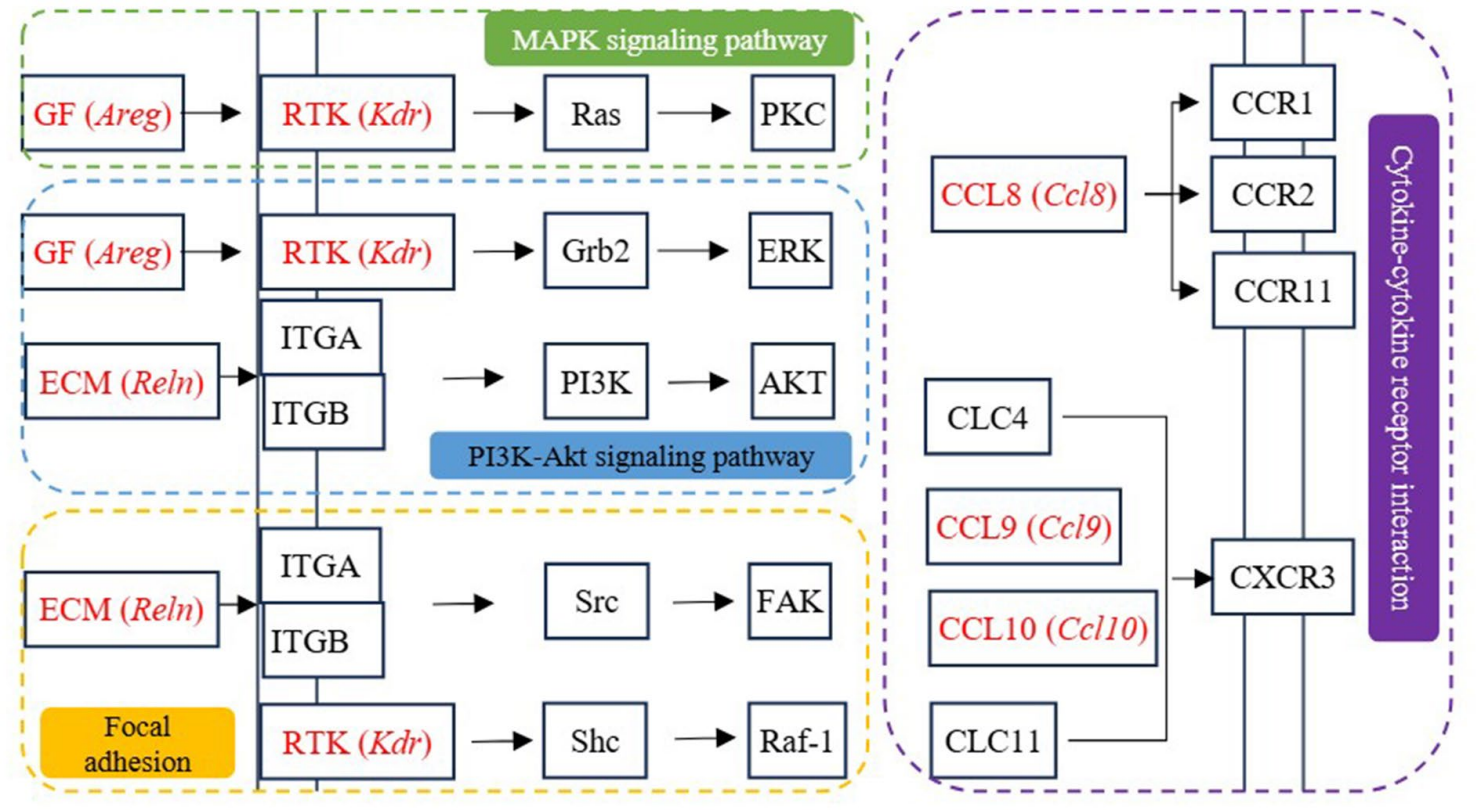
The signaling pathways involved in differentially expressed genes co-regulated by CTRL *vs* LPS and NFS *vs* LPS groups.

A parallel analysis of CTRL *vs* LPS and NFS *vs* LPS comparisons yielded 38 co-regulated DEGs, with 29 genes exhibiting LPS-induced upregulation reversed by NFS treatment, and 9 genes demonstrating reciprocal regulation (Supplementary Table S6). Among these co-regulated genes, *Cxcl10*, *Kdr*, *Areg*, *Cxcl9*, *Reln*, and *Ccl8*, which involved in cytokine-cytokine receptor interaction, PI3K-Akt signaling pathway, focal adhesion, and MAPK signaling pathway, may be the potential targets for N-Feruloylserotonin to regulate intestinal inflammation (Figure 8).

**Figure 9:**
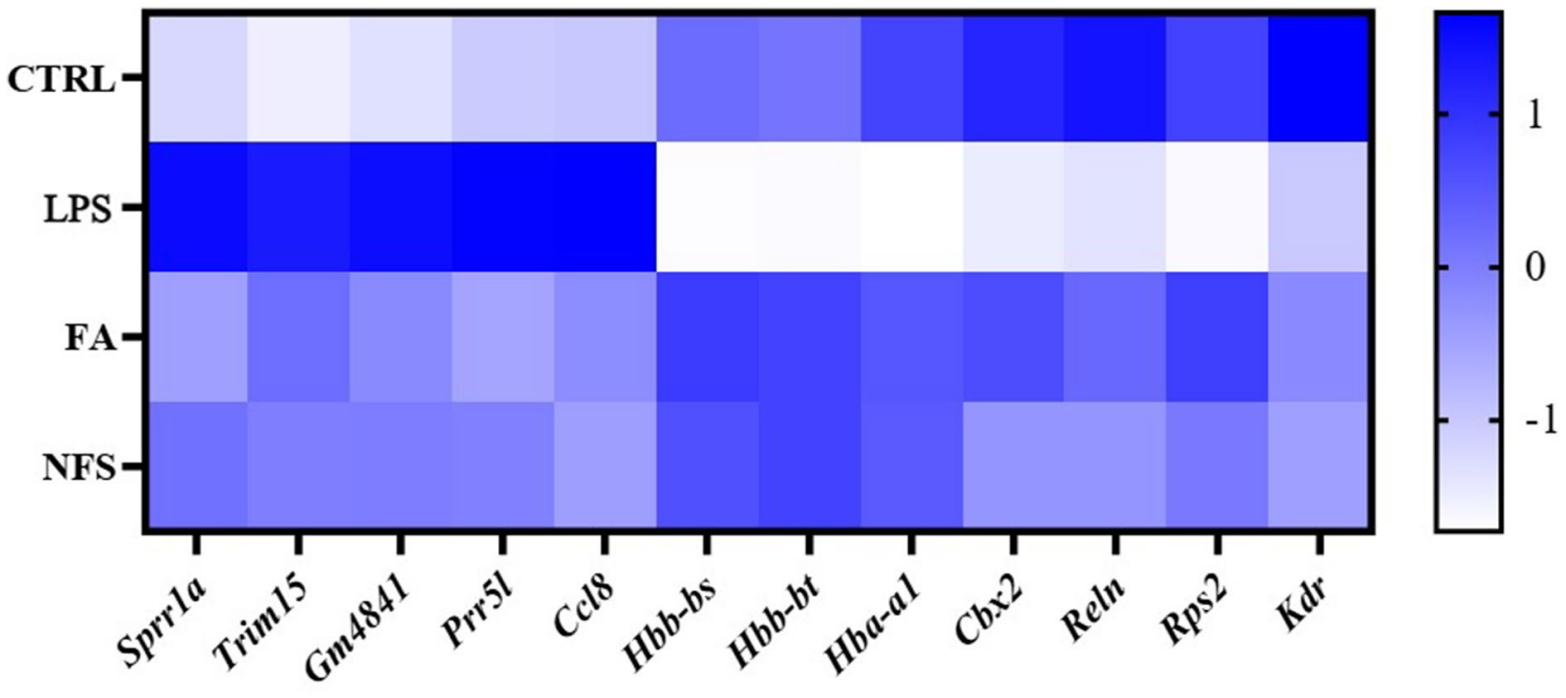
Heatmap of differentially expressed genes.

Heatmaps were constructed to visualize expression patterns of shared co-regulated genes (Figure 9). Among them, *Sprr1a*, *Trim15*, *Gm4841*, *Prr5l*, and *Ccl8* were up-regulated in LPS group and down-regulated in FA and NFS groups. *Hbb-bs*, *Hbb-bt*, *Hba-a1*, *Cbx2*, *Reln*, *Rps2*, and *Kdr* were down-regulated in LPS group and up-regulated in FA and NFS groups.

## 4 Discussion

The effects of ferulic acid and N-Feruloylserotonin on LPS-induced intestinal inflammation were investigated in this study. The results showed that ferulic acid and N-Feruloylserotonin reduced the levels of inflammatory factors and necroptosis pathway genes, increased the expression of genes encoding tight junction proteins, and alleviated the intestinal structural damage caused by LPS. According to the microbial sequencing, ferulic acid and N-Feruloylserotonin promoted the growth of probiotics such as Ruminococcaceae, Akkermansia, Lachnospiraceae, Bifidobacteriaceae, Prevotellaceae and *Roseburia*, and inhibited that of Proteobacteria, Gammaproteobacteria, Enterobacterales and *Bacillus*. Serum metabolites in different groups also changed significantly. LPS reduced metabolites including 1-Naphthalenesulfonic acid, while ferulic acid and N-Feruloylserotonin increased their content. The results of transcriptome sequencing showed that *Sprr1a*, *Trim15*, *Gm4841*, *Prr5l*, and *Ccl8* were up-regulated in LPS group and down-regulated in FA and NFS groups, while *Hbb-bs*, *Hbb-bt*, *Hba-a1*, *Cbx2*, *Reln*, *Rps2*, and *Kdr* were down-regulated in LPS group and up-regulated in FA and NFS groups.

Intestinal epithelial cells construct a barrier that supports nutrient absorption and prevent pathogen invasion. Tight junctions in paracellular spaces are maintained by complex protein–protein interaction networks, and tight junction dysfunction can lead to a variety of local and systemic diseases (Buckley and Turner, 2018). Studies have shown that LPS can increase intestinal permeability and induce the significant down-regulation of *ZO-1* and *Occludin* in cells (He et al., 2022; Liu et al., 2023). In this study, LPS reduced *Claudin 1, Occludin*, and *ZO-1* expression, while ferulic acid and N-Feruloylserotonin increased their expression, indicating that they can effectively reduce intestinal permeability, support intestinal nutrient absorption, and prevent pathogen invasion. *iNOS*, a pro-inflammatory mediator that affects immune system, is mainly expressed by immune cells, including T cells, macrophages, and mature CDs (Xue et al., 2018). It regulates the differentiation and function of immune cells by nitrating key molecules involved in transcription or signaling pathways (Mao et al., 2013). IRF5 is a significant marker for the activation of M1 macrophages, and *iNOS* expressed by macrophages can regulate the balance between M1 and M2 macrophages by modifying it (Ischiropoulos and Gow, 2005). In contrast, CD206 and Arg-1 are specific markers of M2 macrophages, which can defend against pathogens, eliminate apoptotic cells, reduce inflammation and promote wound healing (Rőszer, 2015). According to this study, LPS induced the expression of *iNOS* and reduced that of *CD206* and *Arg-1*, while ferulic acid and N-Feruloylserotonin reversed their expression, indicating that LPS aggravated inflammation, and these two natural active substances effectively alleviated inflammation.

A variety of bacterial communities are colonized in the gut, which affect the functions of the host. The diversity of gut microbiome is an indispensable part of its resilience, the diversified microbiome can resist the invasion of pathogens and ensure the stability of intestinal microecology (Cullen et al., 2023). In this study, LPS significantly reduced Shannon index, Simpson index, Chao1 index, ACE index, and PD-whole-tree index of mice, while ferulic acid and N-Feruloylserotonin significantly increased the α diversity of colonic microorganisms in mice, which contributed to the stability of intestinal microecology in mice. The intestinal microbiota maintains the host mucosal immune system and regulates intestinal homeostasis by interacting with the intestinal mucosa. Typical intestinal bacteria found in healthy individuals include Bacteroidaceae, Clostridiaceae, Prevotellaceae, Eubacteriaceae, Ruminococcaceae, Bifidobacteriaceae, Lactobacillaceae, Saccharomycetaceae, and Methanobacteriaceae (Jackson and Theiss, 2020). Among them, Bacteroidaceae, Prevotellaceae, Ruminococcaceae and Bifidobacteriaceae were significantly up-regulated by Ferulic acid and N-Feruloylserotonin in this study. The members of Bacteroidaceae are the main members of the intestinal bacteria, which can not only degrade complex polysaccharides to provide energy for the colon, but also participate in a variety of important metabolic activities of the body (Zafar and Saier, 2021). Prevotellaceae and Ruminococcaceae are potential probiotics, which are closely related to metabolism and health, and have the function of relieving inflammation (Ortega-Santos and Whisner, 2019; Feng et al., 2022). Bifidobacteriaceae contributes to the absorption of sugar, minerals and nutrients, as well as the synthesis of vitamins. It exerts anti-inflammatory properties by affecting essential fatty acids, and the reduction in its abundance will negatively affect the intestinal epithelial barrier function (Hanifi et al., 2021). Microbial metabolites, the messengers of colonic epithelial cells and immune cells, can affect metabolism, epigenetic modification and gene expression. SCFAs are the most studied microbial metabolites, which provide energy to colonic epithelial cells and contribute to maintain colonic homeostasis (He et al., 2024). In this study, Ruminococcaceae, Muribaculaceae, Akkermansia, Lachnospiraceae, Bifidobacteriaceae, Prevotellaceae, *Roseburia*, *Blautia*, and *Butyricicoccu*s, which can produce SCFAs, were significantly increased in the FA and NFS groups. Proteobacteria contains many pathogenic bacteria, and the increase of its abundance will lead to imbalance among different bacterial species, increase of intestinal permeability and disorder of intestinal microbial community, leading to aggravation of inflammation (Cuesta et al., 2022). Gammaproteobacteria, Enterobacterales and *Escherichia Shigella* belong to Proteobacteria, which were increased significantly in the LPS group and down-regulated significantly by ferulic acid and N-Feruloylserotonin. The above results showed that ferulic acid and N-Feruloylserotonin can alleviate intestinal inflammation by regulating the intestinal flora of mice, they can increase the abundance of beneficial bacteria and reduce that of pathogenic bacteria.

According to the serum metabolomics, some metabolites beneficial to the health of the body were down-regulated in the LPS group. For example, Idebenone is an antioxidant that can effectively inhibit lipid peroxidation in brain tissue and protect cells from oxidative damage (Yan et al., 2018). Phylloquinone belongs to vitamin K, and its most well-known function is to act as a cofactor that activates vitamin K-dependent coagulation factors. (24R)-24-fluoro-1,25-dihydroxyvitamin D2 is a metabolite of vitamin D2. Vitamin D is the precursor of hormones, which plays a key role in regulating calcium and phosphate metabolism, thereby maintaining normal function of bone (Wilson et al., 2017). PE(22:1(11Z)/22:1(11Z)), PE(20:0/22:1(13Z)) and PE(22:4(7Z,10Z,13Z,16Z)/17:0) are phosphatidylethanolamines, which are the second most abundant phospholipids in mammalian cells with strong biological activity. They can act as lipid chaperones to help certain membrane proteins fold, contribute to the initiation of autophagy, and act as receptors for host defense peptides to promote their antibacterial activity (Pohl and Jovanovic, 2019). DG(14:0/19:0/0:0), which belongs to diacylglycerol, is a lipid second messenger that affects the proliferation, survival and intracellular signal transduction of mammalian cells, and exerts many biological functions through protein kinase C, other effector isozymes, and small GTPase regulatory proteins (Cooke and Kazanietz, 2022). PG(12:0/12:0), PG(P-16:0/12:0), and PG(22:1(11Z)/0:0) are phosphatidylglycerols, which can regulate the function of keratinocytes and inhibit the occurrence and development of inflammation (Xie et al., 2018). The promotion of these metabolites by ferulic acid and N-Feruloylserotonin indicated the potential role of these two substances in maintaining body health. In addition, the increase of some harmful substances will have a negative impact on the health of mice. For example, Isoreserpine belongs to alkaloids, which induce endothelial injury and pulmonary hypertension by targeting extracellular calcium-sensitive receptors, as well as acute cerebrovascular disease, astrocytic proliferation and neuronal degeneration associated with behavioral changes in rats (Silva et al., 2023). The increase of Isoreserpine in LPS group indicated the aggravation of intestinal inflammation in mice, while its down-regulation in FA and NFS groups indicated the protective effect of ferulic acid and N-Feruloylserotonin on the health of mice.

LPS is the main structural component of the outer membrane of most Gram-negative bacteria, which can stimulate the immune system and induce a series of pathological states in the body (Kell and Pretorius, 2015). LPS-induced signal transduction processes include intracellular signal transduction directly activated by TLR4 receptor complex and continuous induction of indirect autocrine and paracrine signal transduction events (Bode et al., 2012). Liu et al. used LPS to induce mastitis in dairy cows, and the results found that the DEGs between the CON group and the LPS-treated group were mainly enriched in NF-κB signaling pathway, IL-17 signaling pathway, and cytokine-cytokine receptor interaction, which was consistent with the results of this study (Liu et al., 2024). NF-κB and MAPK signaling pathway are involved in LPS-mediated TNF-α secretion. There is also evidence that the crosstalk between MAPK pathway and signal transduction mediated by STAT 3 forms a critical axis continuously activated by LPS, which is critical for the induction and spread of inflammatory macrophage responses (Bode et al., 2012). As an important intracellular signaling pathway, PI3K/Akt regulates many cellular processes, including cancer progression, cell proliferation, metabolism and survival (Li et al., 2008). PI3K phosphorylation activates Akt, which has a variety of biological functions and activates downstream protein molecules (Xie et al., 2019). Antigen processing and presentation are the basis of adaptive immunity. The major histocompatibility complex (MHC) molecules responsible for antigen presentation are MHCI and MHCII molecules, which present antigen peptides to CD8 T cells and CD4 T cells, respectively (Pishesha et al., 2022). Cytokines play a biological role by binding to the corresponding cytokine receptors on the cell surface. LPS induces the production of cytokines in immune cells, including TNF, IL-1β, IFN-γ and chemokines (Cavaillon, 2018). The combination of cytokines and their receptors initiates complex intracellular molecular interactions, which ultimately lead to changes in cell gene transcription and coordinate the immune response. Moreover, the Ras and Rap1 signaling pathway are also important signaling mechanisms in cells and are involved in many biological processes, such as cell growth, differentiation, apoptosis and metabolism (Kosuru and Chrzanowska, 2020; Sadeghi Shaker et al., 2023). Therefore, according to the results of transcriptome sequencing, genes involved in the above pathways and regulated by ferulic acid, including *Pik3cd*, *Prkca*, *Prkcb*, *Ccnd1*, *Cdkn1a*, *Plcg2*, *Rac2*, *H2-Oa*, *H2-DMb2*, *Tgfa*, *Cxcr4*, *Cxcl12*, *Il2rg*, *Pdgfc*, *Fgfr3*, *Ticam1*, *Irak4*, *Traf6*, and *Lat*, may be potential targets for ferulic acid to alleviate intestinal inflammation. And *Cxcl10*, *Kdr*, *Areg*, *Cxcl9*, *Reln*, and *Ccl8* may be potential targets for N-Feruloylserotonin to alleviate intestinal inflammation.

In addition, some genes shared by the co-regulated genes may also be potential targets for two substances to alleviate intestinal inflammation in mice. Among them, *Sprr1a* encodes small proline rich protein 1A. The knockout of *Sprr1a* in mice can improve cardiac dysfunction after myocardial infarction (Kawaguchi et al., 2023), and the expression of *Sprr1a* in colorectal cancer tissues is significantly increased, which may be used as a potential biomarker for the prognosis of colon cancer (Deng et al., 2020). Hemoglobin is considered to be an iron-containing protein essential for oxygen transport in mammalian blood (Gell, 2018). *Hbb-bs*, *Hbb-bt* and *Hba-al* are genes encoding hemoglobin, which are up-regulated by ferulic acid and N-Feruloylserotonin, helping to maintain the normal functioning of life. Vascular endothelial growth factor (VEGF) is the main growth factor of endothelial cells. The elevated gene *Kdr* in the FA and NFS groups encodes a receptor for VEGF, which is called a kinase insert domain receptor, a key regulator of angiogenesis, and the major mediator of VEGF-induced endothelial proliferation, survival and migration (Boyer, 2002). Notably, the expression of key genes in necroptosis, including *Rip1*, *Rip3*, and *Mlkl*, were also detected in this study. RIP1/RIP3/MLKL pathway is a classical regulatory pathway of necroptosis (Vandenabeele et al., 2010). Under the stimulation of ischemia/reperfusion, inflammation and oxidative stress, RIP1 and RIP3 form a complex through the RIP terminal interaction motif, which then induce the activation and translocation of MLKL, and finally lead to cell lysis (Liu et al., 2019). The activation of caspase-1 and NLRP3 mediated by MLKL, and the secretion of pro-inflammatory cytokine IL-1β are the main determinants of necroptosis-derived inflammatory signals (Conos et al., 2017). The assembly of NLRP3 inflammasome leads to the activation of caspase-1, the release of IL-1β and IL-18, and the cleavage of gasdermin D, thereby promoting cell death and aggravating the occurrence and development of inflammatory diseases (Xu and Núñez, 2023). In this study, LPS increased the expression of *Rip1*, *Rip3*, *Mlkl*, and *IL-1β*, indicating the activation of necroptosis pathway, while ferulic acid and N-Feruloylserotonin inhibited the activation of necroptosis and alleviated intestinal inflammation in mice. Therefore, ferulic acid and N-Feruloylserotonin may alleviate inflammation and maintain health by inhibiting necroptosis.

## 5 Conclusion

**Figure 10:**
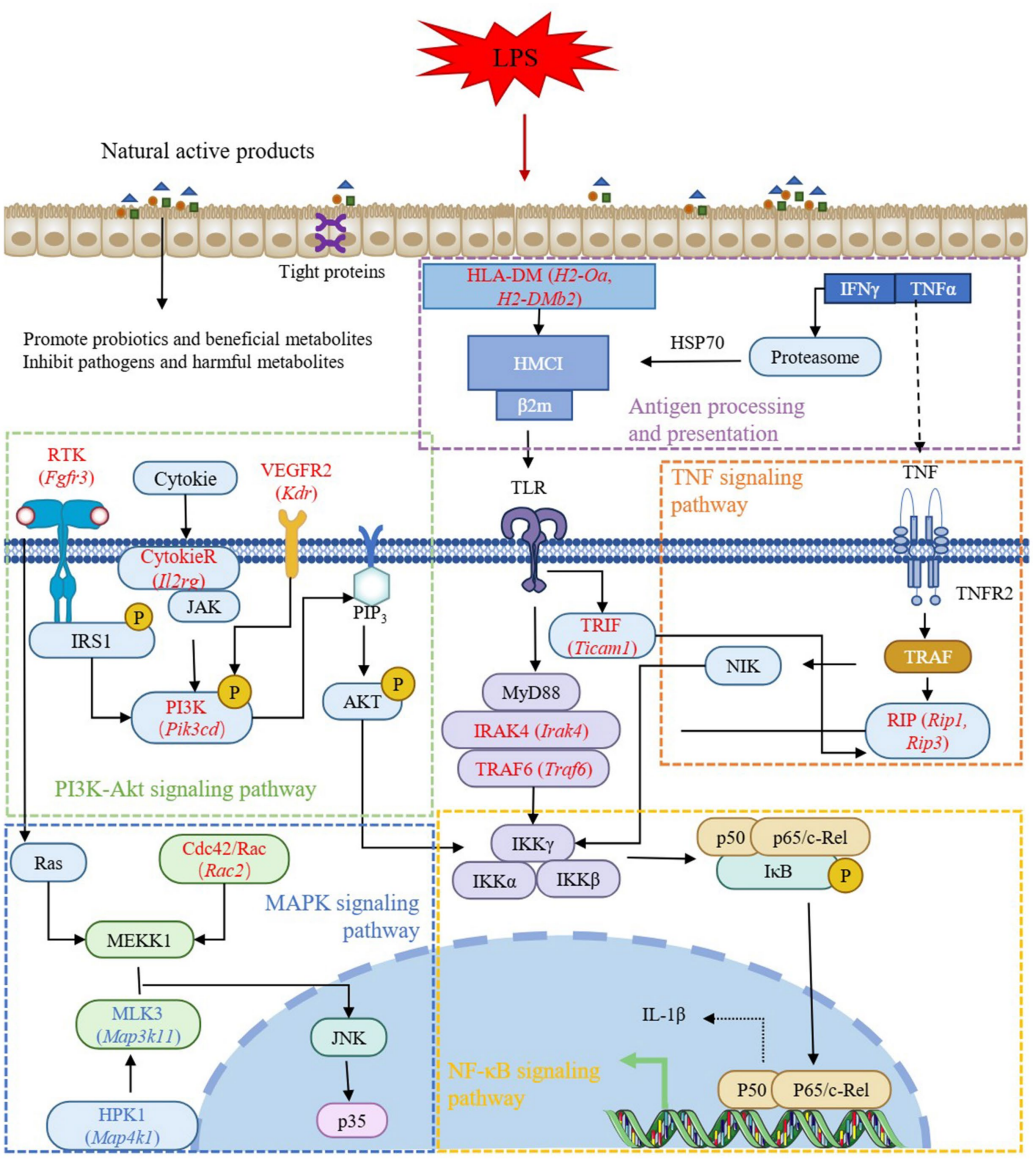
Potential mechanisms of natural active products regulating intestinal inflammation.

Natural active products can mitigate LPS-induced intestinal injury in mice, they may alleviate the intestinal inflammation by regulating antigen processing and presentation, NF-κB signal pathway, MAPK signal pathway and PI3K-Akt signal pathway, among which *Pik3cd*, *H2-DMb1*, *H2-Oa*, *Kdr*, *Fgfr3*, *Il1r2*, *Rac*, *Irak4*, *Traf6*, *Ticam1*, *Rip1*, and *Rip3* are potential targets (Figure 10).

## Data Availability

The data supporting this article have been included as part of the Supplementary material. The 16S rRNA gene raw sequence data and RNA-seq raw sequence data were deposited into the NCBI Sequence Read Archive (SRA) database under the accession numbers PRJNA1237577 and PRJNA1240033, respectively.
