# Supplementary material for: Ferulic acid and N-Feruloylserotonin ameliorate LPS-induced intestinal inflammation via modulation of gut microbiota, metabolome, and transcriptome
Source: Front Microbiol. 2025 Jul 22;16:1597774. doi: 10.3389/fmicb.2025.1597774 (PMC12321762; doi:10.3389/fmicb.2025.1597774)
Supplement: Supplementary file 1 [file Supplementary_file_1.docx]

**Supplementary Table 1** Detailed composition and source of diet of mice

| Main raw materials | |
| --- | --- |
| Protein | Brewer's yeast powder, fish meal, soybean meal |
| Fat | Plant oil |
| Fiber | Wheat bran, Alfalfa meal |
| Carbohydrate | Wheat, corn |
| Minerals | Manganese, stone powder, calcium bicarbonate, iron, zinc, copper, etc. |
| Vitamins | Vitamin A, B1, B6, D, E, pantothenic acid, etc. |

**Supplementary Table 2** Nutrient content of rat diet

| Nutritional composition | Content (%) |
| --- | --- |
| Moisture | 9.2 |
| Crude protein | 19.6 |
| Crude Fiber | 4.5 |
| Crude fat | 5.6 |
| Nitrogen-free Extract | 54.8 |
| Calcium | 1.0 |
| Ash | 6.3 |
| Lysine | 0.92 |
| Phosphorus | 0.8 |
| Methionine and Cystine | 0.63 |

**Supplementary Table 3** qPCR primers

| Primer |  | 5'→3' Sequence |
| --- | --- | --- |
| *β-actin* | Forward | TGTCCACCTTCCAGCAGATGT |
|  | Reverse | AGCTCAGTAACAGTCCGCCTAGA |
| *Rip1* | Forward | TCCTTAGAGGAGGACCAGCG |
|  | Reverse | GGAGTTCGGTGCTGAAGTGG |
| *Rip3* | Forward | CTCCGTGCCTTGACCTACTG |
|  | Reverse | TCACCAGAGGAACCGCATAAC |
| *Mlkl* | Forward | GCGTTGGCCCAAATTTGACT |
|  | Reverse | GGTCTTCTGCCTCGTTGACA |
| *IL-1β* | Forward | TGCCACCTTTTGACAGTGATG |
|  | Reverse | AAGGTCCACGGGAAAGACAC |
| *iNOS* | Forward | GAGCAACTACTGCTGGTGGT |
|  | Reverse | CGATGTCATGAGCAAAGGCG |
| *CD206* | Forward | GTGGAGTGATGGAACCCCAG |
|  | Reverse | CTGTCCGCCCAGTATCCATC |
| *Arg-1* | Forward | TGAGGAAAGCTGGTCTGCTG |
|  | Reverse | AGCCAGCTCTTCATTGGCTT |
| *ZO-1* | Forward | GATCCCTGTAAGTCACCCAGA |
|  | Reverse | CTCCCTGCTTGCACTCCTATC |
| *Occludin* | Forward | TTGAAAGTCCACCTCCTTACAGA |
|  | Reverse | CCGGATAAAAAGAGTACGCTGG |
| *Claudin 1* | Forward | GGGGACAACATCGTGACCG |
|  | Reverse | AGGAGTCGAAGACTTTGCACT |

**Supplementary Table 4** Pathways enrichment score and *P*-values

| Enriched Pathways | Enrichment score | *P*-values |
| --- | --- | --- |
| CTRL vs LPS |  |  |
| Focal adhesion | 1.85 | 3.54E-15 |
| Salmonella infection | 1.69 | 4.11E-12 |
| Axon guidance | 1.77 | 9.27E-11 |
| AGE-RAGE signaling pathway in diabetic complications | 2.06 | 1.81E-10 |
| TNF signaling pathway | 1.93 | 2.34E-10 |
| Prion disease | 1.61 | 2.83E-10 |
| MAPK signaling pathway | 1.55 | 7.66E-10 |
| Proteoglycans in cancer | 1.66 | 1.90E-09 |
| Parkinson disease | 1.59 | 4.88E-09 |
| Regulation of actin cytoskeleton | 1.6 | 7.00E-09 |
| Endocytosis | 1.51 | 8.12E-09 |
| Proteasome | 2.35 | 1.39E-08 |
| Pathways in cancer | 1.35 | 7.83E-08 |
| Huntington disease | 1.47 | 1.42E-07 |
| Alzheimer disease | 1.4 | 3.63E-07 |
| Cholinergic synapse | 1.76 | 4.80E-07 |
| Adherens junction | 1.88 | 4.91E-07 |
| MicroRNAs in cancer | 1.61 | 5.02E-07 |
| Toxoplasmosis | 1.78 | 5.14E-07 |
| Non-alcoholic fatty liver disease | 1.62 | 1.93E-06 |
| FA vs LPS |  |  |
| Oxidative phosphorylation | 2.64 | 2.04E-14 |
| Non-alcoholic fatty liver disease | 2.56 | 3.09E-14 |
| Prion disease | 2.05 | 9.24E-13 |
| Thermogenesis | 2.08 | 1.89E-11 |
| Parkinson disease | 1.92 | 1.27E-09 |
| Pathways in cancer | 1.47 | 5.04E-07 |
| Alzheimer disease | 1.57 | 9.95E-07 |
| Huntington disease | 1.64 | 1.24E-06 |
| MAPK signaling pathway | 1.61 | 2.57E-06 |
| Sulfur metabolism | 5.06 | 3.00E-06 |
| Glycerolipid metabolism | 2.42 | 3.80E-06 |
| Leukocyte transendothelial migration | 2 | 4.98E-06 |
| Salmonella infection | 1.65 | 5.46E-06 |
| Fatty acid degradation | 2.47 | 8.34E-06 |
| Tight junction | 1.7 | 1.69E-05 |
| Focal adhesion | 1.64 | 2.62E-05 |
| Phosphatidylinositol signaling system | 1.98 | 2.73E-05 |
| Carbon metabolism | 1.79 | 3.76E-05 |
| VEGF signaling pathway | 2.25 | 5.38E-05 |
| Peroxisome | 1.94 | 7.39E-05 |
| NFS vs LPS |  |  |
| TNF signaling pathway | 4.55 | 0.000871 |
| Malaria | 5.28 | 0.000947 |
| ECM-receptor interaction | 4.23 | 0.002965 |
| Small cell lung cancer | 4.23 | 0.006587 |
| Toxoplasmosis | 3.74 | 0.010883 |
| Salmonella infection | 2.51 | 0.014472 |
| Cytosolic DNA-sensing pathway | 4.23 | 0.014804 |
| Viral protein interaction with cytokine and cytokine receptor | 3.56 | 0.026056 |
| Complement and coagulation cascades | 3.1 | 0.040203 |
| Human papillomavirus infection | 1.94 | 0.043375 |
| Insulin resistance | 2.99 | 0.04491 |
| Protein digestion and absorption | 2.91 | 0.048635 |
| Toll and Imd signaling pathway | 16.91 | 0.05778 |
| Focal adhesion | 2.14 | 0.062164 |
| Mucin type O-glycan biosynthesis | 4.83 | 0.064133 |
| Pancreatic secretion | 2.58 | 0.069731 |
| Hepatitis B | 2.22 | 0.07483 |
| Toll-like receptor signaling pathway | 2.47 | 0.079298 |
| Neomycin, kanamycin and gentamicin biosynthesis | 12.08 | 0.079953 |
| HIF-1 signaling pathway | 2.42 | 0.084315 |

**Supplementary Table 5** The main differential genes co-regulated by CTRL *vs* LPS and FA *vs* LPS groups

| Gene name | Average FPKM (CTRL) | Average FPKM (LPS) | Average FPKM (FA） | Log_2_ FC (CTRL *vs* LPS） | Regulated | Log_2_ FC (FA *vs* LPS) | Regulated |
| --- | --- | --- | --- | --- | --- | --- | --- |
| *Reg3b* | 5.54 | 8250.45 | 27.49 | 11.05 | up | -8.71 | down |
| *Reg3g* | 6.63 | 5382.72 | 86.63 | 10.27 | up | -6.44 | down |
| *Bcl2l15* | 0.08 | 18.77 | 2.49 | 7.80 | up | -3.52 | down |
| *Apol10a* | 0.49 | 59.74 | 3.88 | 7.59 | up | -4.01 | down |
| *Slc13a1* | 0.39 | 46.47 | 0.89 | 7.16 | up | -6.25 | down |
| *Cyp2c65* | 0.82 | 59.87 | 0.49 | 6.68 | up | -7.41 | down |
| *Mal* | 1.70 | 123.04 | 2.30 | 6.62 | up | -6.26 | down |
| *Sptssb* | 0.96 | 122.09 | 0.63 | 6.58 | up | -8.02 | down |
| *Sprr1a* | 6.40 | 271.76 | 80.36 | 5.88 | up | -2.19 | down |
| *Slc3a1* | 0.62 | 32.09 | 1.95 | 5.85 | up | -4.57 | down |
| *Slc10a2* | 0.90 | 29.29 | 1.22 | 5.59 | up | -5.11 | down |
| *Car1* | 164.06 | 4796.43 | 296.96 | 5.33 | up | -4.52 | down |
| *Cyp4f14* | 0.48 | 15.42 | 1.14 | 5.19 | up | -4.31 | down |
| *Tmigd1* | 3.96 | 148.42 | 8.61 | 5.18 | up | -4.56 | down |
| *Slc51a* | 1.07 | 27.39 | 1.05 | 4.99 | up | -5.09 | down |
| *Slc9a3* | 5.60 | 173.82 | 44.04 | 4.97 | up | -2.46 | down |
| *Oas3* | 1.75 | 34.20 | 11.20 | 4.87 | up | -2.10 | down |
| *Plet1* | 25.97 | 509.41 | 124.68 | 4.86 | up | -2.41 | down |
| *Prkg2* | 0.23 | 4.33 | 0.25 | 4.54 | up | -4.62 | down |
| *Hkdc1* | 0.26 | 5.03 | 0.27 | 4.54 | up | -4.79 | down |
| *Isx* | 1.82 | 47.18 | 2.94 | 4.52 | up | -4.58 | down |
| *Apol9b* | 2.69 | 40.07 | 13.61 | 4.48 | up | -2.02 | down |
| *Fabp2* | 15.82 | 279.52 | 10.48 | 4.34 | up | -5.25 | down |
| *Osr2* | 1.40 | 20.04 | 1.53 | 4.26 | up | -4.30 | down |
| *Cyp2c55* | 20.43 | 307.42 | 26.61 | 4.15 | up | -4.05 | down |
| *Akr1c18* | 0.49 | 6.87 | 0.88 | 4.15 | up | -3.43 | down |
| *Erich4* | 0.67 | 8.03 | 0.85 | 4.08 | up | -3.79 | down |
| *Ces1g* | 0.60 | 7.46 | 1.10 | 4.05 | up | -3.33 | down |
| *Ak4* | 2.51 | 35.02 | 9.89 | 4.04 | up | -2.34 | down |
| *Emp1* | 22.06 | 258.62 | 25.77 | 3.95 | up | -3.77 | down |
| *Mall* | 12.81 | 130.56 | 30.07 | 3.86 | up | -2.63 | down |
| *1810065E05Rik* | 73.87 | 1002.33 | 76.23 | 3.82 | up | -4.21 | down |
| *Gp1bb* | 0.49 | 5.35 | 0.47 | 3.60 | up | -3.94 | down |
| *Aldh1a1* | 24.28 | 219.89 | 16.00 | 3.57 | up | -4.35 | down |
| *Ido1* | 5.55 | 40.27 | 10.95 | 3.31 | up | -2.12 | down |
| *Ceacam2* | 118.62 | 757.37 | 157.10 | 3.24 | up | -2.68 | down |
| *Prr5l* | 0.63 | 4.07 | 1.29 | 3.18 | up | -2.14 | down |
| *Mfsd6l* | 0.40 | 2.72 | 0.60 | 3.17 | up | -2.76 | down |

| Gene name | Average FPKM (CTRL) | Average FPKM (LPS) | Average FPKM (FA） | Log_2_ FC (CTRL *vs* LPS） | Regulated | Log_2_ FC (FA *vs* LPS) | Regulated |
| --- | --- | --- | --- | --- | --- | --- | --- |
| *Dpep1* | 9.21 | 72.55 | 10.70 | 3.11 | up | -3.24 | down |
| *Muc3a* | 11.05 | 59.57 | 15.35 | 3.03 | up | -2.54 | down |
| *Gata3* | 0.30 | 1.63 | 0.45 | 2.97 | up | -2.22 | down |
| *Slc35d1* | 3.44 | 18.76 | 5.66 | 2.94 | up | -2.29 | down |
| *Tmem238l* | 11.06 | 60.23 | 21.01 | 2.91 | up | -2.02 | down |
| *Fam162a* | 77.66 | 402.90 | 100.06 | 2.91 | up | -2.56 | down |
| *Ces1f* | 2.12 | 11.38 | 2.95 | 2.88 | up | -2.53 | down |
| *Fa2h* | 6.61 | 37.63 | 10.47 | 2.88 | up | -2.31 | down |
| *Pla2g12b* | 1.58 | 8.76 | 2.96 | 2.88 | up | -2.19 | down |
| *Slc16a5* | 4.20 | 19.61 | 4.62 | 2.86 | up | -2.56 | down |
| *Rhbg* | 0.64 | 3.18 | 0.94 | 2.82 | up | -2.31 | down |
| *Apol6* | 3.38 | 16.80 | 5.64 | 2.79 | up | -2.11 | down |
| *Pax8* | 0.50 | 2.48 | 0.82 | 2.77 | up | -2.10 | down |
| *Chn2* | 2.08 | 9.34 | 3.31 | 2.72 | up | -2.04 | down |
| *Ifit1bl2* | 1.12 | 6.10 | 1.69 | 2.71 | up | -2.32 | down |
| *Chp2* | 6.97 | 38.76 | 5.23 | 2.69 | up | -3.43 | down |
| *Ggt1* | 0.52 | 2.51 | 0.86 | 2.67 | up | -2.12 | down |
| *Ugt1a1* | 21.15 | 85.86 | 14.96 | 2.61 | up | -3.00 | down |
| *Btnl2* | 1.31 | 5.33 | 0.71 | 2.61 | up | -3.42 | down |
| *Agpat2* | 14.21 | 60.45 | 20.62 | 2.60 | up | -2.06 | down |
| *Prom1* | 14.28 | 66.37 | 15.21 | 2.60 | up | -2.65 | down |
| *Edn3* | 2.05 | 8.64 | 0.70 | 2.55 | up | -4.16 | down |
| *Gpat3* | 1.67 | 7.95 | 1.96 | 2.54 | up | -2.44 | down |
| *H2-T3* | 2.71 | 11.71 | 2.44 | 2.54 | up | -2.82 | down |
| *Espn* | 4.26 | 22.86 | 3.79 | 2.54 | up | -2.90 | down |
| *Cdx2* | 7.20 | 34.25 | 4.99 | 2.45 | up | -3.31 | down |
| *Tpm3-rs7* | 6.98 | 28.18 | 4.48 | 2.45 | up | -3.02 | down |
| *Ces2a* | 8.17 | 27.61 | 7.61 | 2.41 | up | -2.57 | down |
| *Mettl7b* | 21.52 | 83.13 | 13.37 | 2.34 | up | -3.19 | down |
| *Papss2* | 23.20 | 80.00 | 21.45 | 2.25 | up | -2.44 | down |
| *Sult2b1* | 12.04 | 43.12 | 12.44 | 2.25 | up | -2.28 | down |
| *Pcsk5* | 0.45 | 1.30 | 0.48 | 2.24 | up | -2.14 | down |
| *Guca2a* | 275.39 | 1040.61 | 334.93 | 2.19 | up | -2.15 | down |
| *Xpnpep2* | 1.51 | 4.90 | 1.08 | 2.18 | up | -2.72 | down |
| *Cyp2d26* | 4.10 | 12.09 | 3.89 | 2.17 | up | -2.26 | down |
| *Guca2b* | 14.02 | 43.26 | 12.34 | 2.17 | up | -2.33 | down |
| *Gcnt3* | 6.61 | 22.30 | 6.21 | 2.16 | up | -2.38 | down |
| *Eps8l2* | 11.06 | 35.30 | 10.85 | 2.08 | up | -2.19 | down |
| *Clec2e* | 11.25 | 38.47 | 6.64 | 2.07 | up | -3.06 | down |

| Gene name | Average FPKM (CTRL) | Average FPKM (LPS) | Average FPKM (FA） | Log_2_ FC (CTRL *vs* LPS） | Regulated | Log_2_ FC (FA *vs* LPS) | Regulated |
| --- | --- | --- | --- | --- | --- | --- | --- |
| *Klhl6* | 9.08 | 1.27 | 8.13 | -2.05 | down | 2.01 | up |
| *Trabd2b* | 0.44 | 0.07 | 0.53 | -2.07 | down | 2.28 | up |
| *Fcrl1* | 13.56 | 1.77 | 13.84 | -2.11 | down | 2.19 | up |
| *Tcf7* | 7.23 | 0.88 | 5.68 | -2.32 | down | 2.10 | up |
| *Cnr2* | 2.19 | 0.24 | 1.48 | -2.42 | down | 2.00 | up |
| *Pthlh* | 2.62 | 0.31 | 2.06 | -2.44 | down | 2.07 | up |
| *Cyp2d12* | 6.88 | 0.97 | 6.68 | -2.49 | down | 2.12 | up |
| *Pax5* | 5.25 | 0.53 | 3.49 | -2.51 | down | 2.02 | up |
| *Cd19* | 18.43 | 1.79 | 11.71 | -2.54 | down | 2.01 | up |
| *Wdfy4* | 2.61 | 0.25 | 1.76 | -2.62 | down | 2.17 | up |
| *Vpreb3* | 9.93 | 0.89 | 7.14 | -2.63 | down | 2.27 | up |
| *Lyz1* | 22.16 | 2.10 | 14.57 | -2.65 | down | 2.16 | up |
| *Slc16a12* | 1.34 | 0.13 | 0.91 | -2.71 | down | 2.24 | up |
| *Blk* | 7.58 | 0.65 | 4.56 | -2.79 | down | 2.14 | up |
| *Rasal3* | 4.31 | 0.39 | 2.34 | -2.82 | down | 2.04 | up |
| *Foxq1* | 2.13 | 0.20 | 1.82 | -2.84 | down | 2.67 | up |
| *Cd37* | 34.39 | 2.91 | 17.66 | -2.86 | down | 2.01 | up |
| *Glycam1* | 63.20 | 5.16 | 48.20 | -2.89 | down | 2.48 | up |
| *Irs1* | 1.24 | 0.11 | 1.68 | -2.91 | down | 3.34 | up |
| *Gjb5* | 5.07 | 0.50 | 2.92 | -2.96 | down | 2.08 | up |
| *Cd79a* | 73.16 | 5.43 | 40.27 | -2.99 | down | 2.25 | up |
| *Siglecg* | 4.29 | 0.30 | 2.15 | -3.05 | down | 2.14 | up |
| *Cd6* | 1.72 | 0.13 | 0.82 | -3.07 | down | 2.12 | up |
| *Fcrla* | 12.66 | 0.85 | 6.40 | -3.07 | down | 2.18 | up |
| *Cd79b* | 55.57 | 3.46 | 28.47 | -3.17 | down | 2.31 | up |
| *Fmo2* | 22.01 | 1.80 | 9.83 | -3.34 | down | 2.08 | up |
| *Btla* | 9.53 | 0.64 | 5.36 | -3.45 | down | 2.68 | up |
| *Chst3* | 2.47 | 0.12 | 1.06 | -3.50 | down | 2.35 | up |
| *H2-Ob* | 14.41 | 0.70 | 6.36 | -3.51 | down | 2.43 | up |
| *H2-Eb2* | 3.27 | 0.16 | 1.98 | -3.51 | down | 2.95 | up |
| *Best2* | 5.31 | 0.33 | 2.74 | -3.52 | down | 2.56 | up |
| *Cr2* | 8.83 | 0.38 | 3.07 | -3.64 | down | 2.27 | up |
| *Gpr174* | 1.82 | 0.08 | 0.71 | -3.72 | down | 2.49 | up |
| *Fcmr* | 24.37 | 0.94 | 9.76 | -3.87 | down | 2.65 | up |
| *Bank1* | 6.44 | 0.25 | 2.70 | -4.01 | down | 2.89 | up |
| *Il22ra2* | 12.42 | 0.40 | 3.22 | -4.35 | down | 2.40 | up |
| *Fcer2a* | 12.39 | 0.28 | 2.14 | -4.65 | down | 2.27 | up |
| *Slc37a2* | 24.04 | 0.39 | 8.33 | -5.57 | down | 3.98 | up |
| *Ermap* | 4.69 | 0.06 | 1.30 | -5.72 | down | 3.93 | up |

**Supplementary Table 6** The main differential genes co-regulated by CTRL *vs* LPS and NFS *vs* LPS groups

| Gene name | Average FPKM (CTRL) | Average FPKM (LPS) | Average FPKM (NFS） | Log_2_ FC (CTRL *vs* LPS） | Regulated | Log_2_ FC (NFS *vs* LPS) | Regulated |
| --- | --- | --- | --- | --- | --- | --- | --- |
| *Cxcl10* | 7.12 | 325.04 | 118.07 | 6.18 | up | -1.70 | down |
| *Ltf* | 0.24 | 10.88 | 2.96 | 6.00 | up | -2.19 | down |
| *Sprr1a* | 6.40 | 271.76 | 139.20 | 5.88 | up | -1.21 | down |
| *Slfn4* | 9.11 | 237.88 | 140.99 | 5.27 | up | -1.03 | down |
| *Trim15* | 8.86 | 178.46 | 98.39 | 4.77 | up | -1.07 | down |
| *Cxcl9* | 4.80 | 76.32 | 33.29 | 4.51 | up | -1.35 | down |
| *Gm4841* | 1.01 | 12.31 | 6.25 | 4.36 | up | -1.22 | down |
| *Gm4951* | 0.87 | 9.63 | 4.65 | 4.13 | up | -1.23 | down |
| *Trib3* | 0.58 | 6.63 | 3.11 | 3.81 | up | -1.24 | down |
| *Urah* | 6.38 | 57.79 | 24.86 | 3.59 | up | -1.37 | down |
| *Wfdc18* | 12.89 | 90.91 | 40.79 | 3.42 | up | -1.49 | down |
| *Prr5l* | 0.63 | 4.07 | 1.90 | 3.18 | up | -1.27 | down |
| *Mx1* | 2.10 | 12.32 | 6.69 | 3.07 | up | -1.06 | down |
| *Ccl8* | 16.91 | 85.92 | 31.54 | 2.80 | up | -1.64 | down |
| *Apol10b* | 0.55 | 2.49 | 1.36 | 2.73 | up | -1.12 | down |
| *Krt36* | 0.87 | 3.81 | 1.37 | 2.65 | up | -1.60 | down |
| *Ly6c1* | 65.14 | 265.56 | 134.94 | 2.53 | up | -1.27 | down |
| *Wfdc17* | 13.73 | 55.12 | 31.75 | 2.46 | up | -1.08 | down |
| *Slfn5* | 4.01 | 14.73 | 7.29 | 2.44 | up | -1.23 | down |
| *Erich3* | 0.27 | 0.94 | 0.44 | 2.31 | up | -1.22 | down |
| *Tac1* | 69.42 | 246.46 | 114.18 | 2.24 | up | -1.34 | down |
| *Slfn1* | 1.37 | 3.64 | 2.15 | 2.03 | up | -1.07 | down |
| *Nlrp10* | 0.50 | 1.45 | 0.70 | 1.99 | up | -1.21 | down |
| *Calhm6* | 2.15 | 5.55 | 3.14 | 1.93 | up | -1.08 | down |
| *Areg* | 7.21 | 18.08 | 9.03 | 1.81 | up | -1.23 | down |
| *Slc17a8* | 0.37 | 0.73 | 0.40 | 1.57 | up | -1.05 | down |
| *Col17a1* | 0.26 | 0.59 | 0.32 | 1.55 | up | -1.01 | down |
| *Amd2* | 1.50 | 2.72 | 0.88 | 1.42 | up | -1.74 | down |
| *Rdh16* | 8.87 | 15.27 | 8.52 | 1.22 | up | -1.06 | down |
| *Hbb-bs* | 50.46 | 16.46 | 57.03 | -1.26 | down | 1.58 | up |
| *Hbb-bt* | 15.56 | 4.77 | 19.63 | -1.37 | down | 1.86 | up |
| *Hba-a1* | 72.81 | 20.17 | 66.85 | -1.48 | down | 1.49 | up |
| *Cbx2* | 0.84 | 0.21 | 0.48 | -1.49 | down | 1.05 | up |
| *Fmo5* | 1.07 | 0.26 | 0.82 | -1.59 | down | 1.43 | up |
| *Reln* | 0.92 | 0.19 | 0.46 | -1.89 | down | 1.07 | up |
| *Rps2* | 38.37 | 5.81 | 28.50 | -1.96 | down | 2.20 | up |
| *Kdr* | 1.88 | 0.22 | 0.57 | -2.64 | down | 1.13 | up |
| *Dbp* | 34.98 | 3.32 | 10.52 | -3.03 | down | 1.54 | up |
